# Pomarius

**Published:** 1875-09

**Authors:** 


### POMARIUS.

There is a new and exceedingly delicate edible, to be shortly introduced by our indefatigable neighbors, the Health Food Co. It is a new and fresh fruit juice, reduced to a jelly-like consistence by careful evaporation. No sugar is added and no gelatine. It is, in fact, simply fresh fruit juice, with its watery constituents evolved by heat. It will keep for years, in an open vessel, and never grows musty or sour, or in any way deteriorates. It is more delicious than currant jelly and more grateful to the stomach than any of the sauces or preserves. It will become a standard in sickness and on the tables of refined persons, as soon as known.

---

An Escharotic.—Carbolic acid is as clearly an escharotic as hot iron or the cautery. Its destructive power over tissues is not apparent as generally used in weak solution, but in anything like concentrated form it burns like the strongest mineral acids. Such is the rather tardy conclusion to which a physician in Medford has arrived. He recommended a lady who was threatened with a felon, to saturate a cloth with carbolic acid and bind her finger in it. She did as directed, and when she looked at her finger the next morning it was black and dead. It is said that the physician, a man nearing middle age, who has practised for several years in Medford, could only say that he had made a mistake. The aid of other medical men was sought and everything was done, but all to no purpose, and she was obliged to have the finger amputated below the second joint. This loss is particularly trying, as she has been considered a fine musician. The husband threatens the physician with prosecution.

---

If you wish to make a nail drive easily and last long without rusting, dip it in melted grease first. This is excellent for fencing and other exposed work.

